# Early-life risk factors predicting growth retardation and mortality in pigs: a multi-criteria approach

**DOI:** 10.1093/jas/skaf402

**Published:** 2025-11-19

**Authors:** Pau Salgado-López, Katelyn N Gaffield, Mike D Tokach, Jaume Coma, Josep Gasa, Mercè Farré, David Solà-Oriol

**Affiliations:** Animal Nutrition and Welfare Service (SNIBA), Department of Animal and Food Science, Autonomous University of Barcelona, Bellaterra, Spain; Department of Animal Sciences and Industry, College of Agriculture, Kansas State University, Manhattan, KS 66506-0201; Department of Animal Sciences and Industry, College of Agriculture, Kansas State University, Manhattan, KS 66506-0201; Vall Companys Group, Lleida, Spain; Animal Nutrition and Welfare Service (SNIBA), Department of Animal and Food Science, Autonomous University of Barcelona, Bellaterra, Spain; Department of Mathematics, Area of Statistics and Operations Research, Autonomous University of Barcelona, Bellaterra, Spain; Animal Nutrition and Welfare Service (SNIBA), Department of Animal and Food Science, Autonomous University of Barcelona, Bellaterra, Spain

**Keywords:** binomial logistic regression, body weight, colostrum intake, growth performance, preweaning survival, swine

## Abstract

Body weight (**BW**) variability throughout the production cycle remains a major challenge for the swine industry, particularly due to the negative impact of slow-growing pigs on production efficiency and batch uniformity. This study aimed to identify early-life risk factors associated with poor postnatal growth and mortality and to develop a multi-criteria predictive model for classifying pigs based on their early growth and survival potential. Data from 2,138 pigs (Pietrain × [Landrace × Yorkshire]) and 1,115 pigs (Pietrain × [Landrace × Large White]), collected from two commercial farms, were analyzed. Pigs were monitored from birth to weaning, with detailed records of farrowing traits, BW, body conformation indicators, colostrum intake (**CI**), and survival outcomes. Body weight on d 7 was the strongest predictor of weaning weight (*R*^2^ > 0.60), highlighting the critical influence of the first week on later growth. Logistic regression models were used to classify pigs as either compromised (defined as dead or alive with a BW below the 15th percentile of the BW distribution on d 7 of life and at weaning) or normal. The classification performance of the competing models, as well as the selection of the final model, was evaluated using the area under the curve (**AUC**) of the receiver operating characteristic curve. Subsequently, the optimal classification threshold was adjusted to balance sensitivity and positive predictive value. The final model, trained on d 7 data, achieved a high AUC (0.910), with BW on d 1, relative BW (**RBW**) on d 1, CI, and sow parity all significantly associated with the probability of being classified as compromised (*P *< 0.05). Each 100 g increase in BW on d 1 was associated with a 27.6% decrease in the odds of being compromised. Similarly, greater RBW on d 1 and CI were linked to a reduced risk. Pigs falling within the 10th percentile for BW on d 1, with low CI and negative RBW on d 1, showed the highest probability of being compromised by d 7. The model’s robustness was confirmed through consistent performance across datasets. Density plots further validated the model, illustrating clear distributional differences between compromised and normal pigs. These findings suggest that a model based on easily measurable birth-related indicators can reliably identify pigs at risk of poor early-life performance. Such a tool holds strong potential for on-farm application to enhance pig management and reduce BW variability at slaughter.

## Teaser

The development of a multi-criteria model based on easily measurable birth-related risk factors, with broad applicability across different pig populations, could enable the swine industry to identify pigs at risk of growth retardation or early mortality. In turn, this could facilitate timely interventions, improve efficiency, and reduce body weight variability at slaughter.

## Introduction

One of the main factors affecting the efficiency of global pig production is the variability in body weight (**BW**) throughout the production cycle. For sanitary reasons, it is recommended to maintain contemporary pigs in the same batch (Maes et al., 2004), making BW variability a limiting factor in optimizing current all-in-all-out swine production systems (Patience et al., 2004). The main contributors to this variability are slow-growing pigs, which are expected to reach slaughter weight later than their faster-growing counterparts (Douglas et al., 2014; He et al., 2016). This subset of pigs has increased in recent decades due to continuous genetic advancements aimed at increasing sow prolificacy and, consequently, the number of pigs produced per sow per year (Rutherford et al., 2013). Calderón Díaz et al. (2017) estimated that, in any given batch, 10% to 15% of pigs are slow-growing pigs.

Based on an epidemiological approach, López-Vergé et al. (2018) suggested that the closer a light pig is to the end of the production cycle, the greater the probability it will be sent to slaughter at a later stage. This is due to the decreasing opportunity to implement effective strategies that enable lighter pigs to catch up to their heavier pen mates, particularly from weaning onward (Blavi et al., 2021). Identifying potential risk factors associated with the survivability and growth performance of these pigs during the early stages of the production cycle is crucial for the timely implementation of effective preventive measures aiming to enhance their overall performance. Previous studies showed that weaning weight (**WW**) has a significant impact on postweaning growth, the number of days required to reach the target slaughter BW, and rates of morbidity and mortality (Wolter and Ellis, 2001; Douglas et al., 2013). Furthermore, Casellas et al. (2024) and Salgado-López et al. (2024) highlighted the substantial contribution of including WW as a prediction factor for subsequent BW in enhancing the performance of different machine learning models used to classify pigs at risk of growth retardation across the different stages of the production cycle until slaughter. Achieving a high BW at weaning is crucial, as changes in pigs’ BW category beyond this point are uncommon (Blavi et al., 2021).

Several studies have identified birth BW as a key indicator of postnatal performance, with pigs born underweight often remaining stunted throughout their life and unable to catch up with their faster-growing counterparts (Gondret et al., 2005; Rehfeldt et al., 2008; Fix et al., 2010). The relationship between birth BW and survivability is also well established in the literature (Van der Lende and De Jager, 1991; Roehe and Kalm, 2000). However, other studies have shown that some pigs with low birth BW can exhibit compensatory growth during early stages (e.g., until the end of lactation), suggesting that birth BW alone may not be the best predictor of subsequent growth performance (Douglas et al., 2013; Montoro et al., 2020). This highlights the potential for targeted management strategies to enhance their lifetime growth.

Many studies have examined additional factors influencing pig growth and survival during the lactation period. Casellas et al. (2024) demonstrated that the probability of being small at weaning is not solely determined by birth BW at the beginning of the period. Other factors such as the difference between an individual pig’s birth BW and the average litter weight, as well as within-litter birth BW variance, also play a role. These findings highlight the importance of cross-fostering for the classification of low BW pigs at weaning. Some authors have suggested that indicators of body conformation, such as body mass index (**BMI**) and ponderal index (**PI**), may serve as better predictors of survivability than birth weight alone (Baxter et al., 2008, 2009; Hales et al., 2013). Other authors have linked pig neonatal vitality to both survival and growth performance during lactation (Herpin et al., 1996; Baxter et al., 2008; Muns et al., 2013). Moreover, all these factors can influence colostrum intake (**CI**), which is critical for postnatal survival and growth up to weaning (Quesnel et al., 2023).

All the studies mentioned above evaluated the individual impact of specific risk factors. To effectively develop early-life management and nutritional strategies for pigs at higher risk of poor growth performance and reduced survivability, the swine industry would benefit from identifying generalizable risk factors associated with compromised pigs. Building on this background, the following sections describe the present study and the work conducted to address these issues. Two distinct pig populations were characterized from birth to weaning, generating two datasets used to 1) identify key factors associated with low growth performance and early-life mortality and 2) characterize different pig populations based on these risk factors, which are critical for predicting early-life mortality or poor growth. The objective of this research was to propose a multi-criteria model, based on the most relevant birth-related risk factors, for general on-farm application. The goal is to improve the future performance of the most vulnerable pigs by focusing on easily measurable on-farm criteria. We hypothesize that integrating multiple birth-related and early-life risk factors into a single multi-criteria model, derived from robust and powerful datasets, would enable accurate prediction of early-life mortality and poor growth, providing a practical tool to identify at-risk pigs and guide timely interventions during the early postnatal period.

## Materials and Methods

The study was conducted with the approval of the Animal Ethics Committee of the Universitat Autònoma de Barcelona (CEEAH2788M2), in accordance with the European Union guidelines for the care and use of animals in research (European Parliament, 2010). Two independent populations of pigs were studied from birth to weaning on two commercial farms in Spain. A summary of each dataset (**DS**) is presented in Table 1. Dataset 1 was collected from 11 consecutive batches on a 1,050-sow farm (Landrace × Yorkshire) with a weekly batch management system. Dataset 2 was collected from 6 consecutive batches on a 600-sow farm (Landrace × Large White) with a 4-wk batch management system.

**Table 1.** Summary of the two datasets analyzed, including the number of pigs, genetic lines, average lactation length, and management practices

Item	Dataset 1	Dataset 2
**Number of pigs, *n***	2,138	1,115
**Data collection**	March 2022–July 2022	January 2024–June 2024
**Pig crossbred line**	(P × [Ld × Y])	(P × [Ld × Lw])
**Sex**	Males and females	Males and females
**Length of lactation, d**	26.1 ± 0.44	21.2 ± 1.25
**Type of management**	Weekly	4 wk
**BW recording**	Birth to weaning	Birth to weaning

Ld = Landrace; Lw = Large white; P = Pietrain; Y = Yorkshire.

### Animals, diets, and housing

In DS 1, 2,138 crossbred pigs (Pietrain × [Landrace × Yorkshire]) from 102 litters were used, whereas in DS 2, 1,115 crossbred pigs (Pietrain × [Landrace × Large White]) from 86 litters were included. In both DS 1 and DS 2, sow parities ranged from 1 to 9, with means ± SD of 3.5 ± 2.09 and 4.4 ± 2.41, respectively.

Sows in both datasets were fed commercial pelleted diets. During gestation, the diets consisted of corn, wheat, and wheat middlings (15.5% crude protein), while during lactation, they were primarily composed of corn, wheat, wheat middlings (15.5% crude protein), and soybean meal. The gestation diets provided 2,200 kcal/kg of feed and 0.500% standardized ileal digestible (**SID**) Lys, whereas the lactation diets supplied 2,412 kcal/kg of feed and 0.804% SID Lys. In DS 1, each female received 2.2 kg of feed per day throughout gestation (from d 30 until placement in the farrowing pens). During the pre-farrowing period, gilts and sows were provided 2.6 and 3.0 kg of feed per day, respectively. Starting the day after farrowing, the daily feed allowance was increased by 400 g per day until d 9 post-farrowing, after which females were fed ad libitum, reaching a maximum intake of up to 9 kg of feed per sow per day. In DS 2, each female also received 2.2 kg of feed per day throughout gestation (from d 30 until placement in the farrowing pens). Before farrowing, gilts and sows received 2.8 kg and 3.2 kg of feed per day, respectively. For gilts, the daily allowance was reduced to 2.6 kg around farrowing and gradually increased to 5 kg/d by d 10 of lactation, reaching 7.5 kg/d at weaning. For sows, the feeding rate was maintained at 3.2 kg/d around farrowing and progressively increased to 6.0 kg/d by d 7, 8.0 kg/d by d 14, and 9.0 kg/d at weaning.

During gestation, sows were housed in groups of 30 to 35 in pens with concrete slatted floors. Five days before the expected farrowing date, sows were moved to the farrowing unit. The farrowing rooms were diffuse ventilated, with a room temperature of approximately 24°C. Artificial lighting was provided from 0600 h to 1400 h in DS 1 and from 0700 h to 1800 h in DS 2. During farrowing, the lights remained switched on until the last monitored sow had finished farrowing, typically around 0100 h or 0200 h. Individual farrowing pens had slatted metal flooring in the sow’s area and slatted plastic flooring in the rest of the pen. During the first days after parturition, a heat lamp was placed above the heat pad.

### Management routines

Cross-fostering was performed within the first 24 h after birth, once it was ensured that all pigs had consumed colostrum. A different protocol was followed on each of the farms under study. In the farm from DS 1, the protocol included the following steps: 1) all litters were equalized to 15 pigs, 2) when litter sizes exceeded 15 pigs, those with the lowest and highest birth weights were fostered to smaller litters, and 3) pigs were cross-fostered only into litters with fewer than 15 pigs on d 1 of life. In the farm from DS 2, the protocol included the following steps: 1) all litters were equalized to a number of pigs equal to or greater than 12, 2) when litter sizes exceeded functional teat count + 1, pigs were fostered to smaller litters, and 3) pigs were cross-fostered only into litters with fewer than 12 pigs on d 1 of life. These protocols aimed to keep the maximum number of pigs with their biological sow. Tail docking and iron injection (Uniferon^®^, Intervet Internation B.V., Boxmeer, The Netherlands) were performed on d 3 after farrowing. Pigs had free access to water, and creep feed (crude protein = 16.91%, net energy = 2,423 kcal/kg, SID Lys = 1.2 g/kg) was offered *ad libitum* starting 10 d before weaning. In DS 1, pigs were weaned 4 wk after farrowing (mean ± SD: 26.1 ± 0.44 d), while in DS 2, pigs were weaned 3 wk after farrowing (mean ± SD: 21.2 ± 1.25 d). Pigs deemed unlikely to survive until weaning due to trauma, disease, or other causes were humanely euthanized by blunt-force trauma. Apart from the recordings made for the study, all animals were managed according to the standard routines of the farms.

### Recordings

All live-born pigs were subjected to measurements of farrowing and morphological traits (Table 2). On the day of birth, prior to litter equalization, each pig was individually identified with an ear tag (LeeO^®^, MSD Animal Health, Spain). Birth order and the time of birth for each pig (stillborn and born alive) were recorded during farrowing supervision. Farrowing traits included birth order and cumulative birth interval, defined as the interval elapsed from the birth of each pig to that of the first pig in the litter. In cases where pigs were cross-fostered, both the date of transfer and the identity of the receiving sow were recorded. Sex, BW, and crown-to-rump length (**CRL**, the supine length of the pig from the crown of the head to the base of the tail) were recorded at birth. Rectal temperature of each pig was measured within the first 30 min after birth. From these measurements, the following indicators of body conformation were calculated: BMI (birth weight (kg)/[CRL (m)]^2^), PI (birth weight (kg)/[CRL (m)]^3^), and relative BW (**RBW**) at birth ([(pig birth weight—mean birth weight of litter)/mean birth weight of litter] × 100) and on d 1 ([(pig BW on d 1—mean weight of litter d 1)/mean weight of litter d 1] × 100). The vitality score at birth was assessed on a 0–3 scale, calculated as the sum of two behavioral variables described by Muns et al. (2013). Udder stimulation: score of 0 if the pig showed no head movements (e.g., no udder stimulation or searching behavior within 30 s), and 1 if head movements were observed. Number of completed circles around enclosure: score of 0 if the pig was unable to rotate its body axis 360° from its initial orientation or walk along the limits of the bucket (55-cm diameter × 60-cm height, solid plastic enclosure, open at the bottom and top); 1 if it could either rotate 360° or walk once along the limits within 30 s; and 2 if it could either rotate 360° or walk along the limits at least twice within 30 s. Pigs were weighed again on d 1 (after cross-fostering), d 7, and at weaning. Body weight at birth and on d 1 were used to calculate 24-h weight gain and to estimate individual CI using the equation developed by Theil et al. (2014). Pig survival throughout the lactation period was monitored and recorded.

**Table 2.** List of additional individual variables evaluated in pigs at birth and on the first day of life, used in the logistic regression analysis of survivability and growth performance

Item	Variables	Measuring unit
**Farrowing traits**	Birth order	*n*
	Cumulative birth interval	min
	Cross-fostering	yes/no
**Pig traits at birth**	Sex	male/female
** Physical characteristics**	Crown-to-rump length	cm
	Body mass index	kg/(m)^2^
	Ponderal index	kg/(m)^3^
	Relative birth weight	%
** Rectal temperature**	Rectal temperature at birth	°C
** Vitality**	Vitality score	U + NCC = Score 0, 1, 2, 3
	Udder stimulation (U)^1^	Score 0, 1
	No. of completed circles around enclosure (NCC)^2^	Score 0, 1, 2
**Pig performance after birth**	BW gain for 24 h after birth	g
	Relative body weight on day 1	%
	Colostrum intake	g/pig
**Pig survival**	Survival within the first week	yes/no
	Survival within the lactation period	yes/no

1 U: score of 0 if the pig showed no head movements (e.g., no udder stimulation or searching behavior within 30 s), and 1 if head movements were observed.

2 NCC: score of 0 if the pig was unable to rotate its body axis 360° from its initial orientation or walk along the limits of the bucket; 1 if it could either rotate 360° or walk once along the limits within 30 s; and 2 if it could either rotate 360° or walk along the limits at least twice within 30 s.

BW = Body weight.

### Calculations and statistical analyses

All calculations and statistical analyses were performed using the open-source software R v.4.4.0 (R Core Team, 2024). Simple linear regression models, implemented via the *lm()* function, were used to identify which early-life growth performance variables (BW on d 1, BW on d 7, and average daily gain (**ADG**) during the first week) best predicted pig WW individually. The coefficient of determination (R^2^) was used to compare the models.

To identify potential risk factors associated with pig short survival and poor postnatal growth during the first week of life and throughout the lactation period, binomial logistic regression analysis (*glm()* function) was performed. A binary (1|0) outcome variable was created, classifying each pig as either compromised (1: dead or alive with a BW below the 15th percentile of the BW distribution at a fixed time point for its respective farm) or normal (0: alive with a BW above the 15th percentile). This classification was applied for both time points: d 7 of age and the end of the lactation period. Percentiles were calculated separately for each farm, as the populations in DS 1 and DS 2 differed. The 15th percentile was selected as the cut-off point to balance between avoiding focusing on extreme values (e.g., outliers) that may arise from using lower percentiles and preventing excessive inclusion of pigs as compromised when using higher percentiles. The final DS used in the models had an unbalanced partition, which is common in cases involving death or compromised pigs, as these are typically minority outcomes. Stillborn pigs or those excluded from the study because they were cross-fostered to off-test sows were not included in the final DS. Moreover, to train the model, edge cases (pigs between the 15th and 16th percentiles) that could complicate the training process were removed and were reinserted once predicted with the selected model.

A subset of variables described in Table 2 (i.e., farrowing traits, pig traits at birth, and pig performance after birth), together with BW on d 1 and sow farrowing traits, were included as predictors in the multiple logistic regression model. Initially, a stepwise backward selection procedure based on the Akaike Information Criterion (**AIC**), where a lower AIC indicates a better model, was applied using the *step()* function to identify potential risk factors for inclusion in the candidate models. Secondly, an ANOVA F-test (using the *anova()* function) for nested models was conducted to confirm whether certain groups of variables were not jointly significant at the 5% level; such variables were then removed. Simultaneously, multicollinearity among predictors was assessed by calculating the Variance Inflation Factor (**VIF**) using the *vif()* function from the car package (Fox and Weisberg, 2019). Variance Inflation Factor values greater than 5 or 10 were considered indicative of moderate and severe multicollinearity, respectively. In such cases, one or more variables with high VIF were considered for exclusion to mitigate multicollinearity.

After selecting candidate models based on multiple criteria (AIC, VIF, and coefficient interpretability), we evaluated the predictive performance of each model to determine the final selection. Once a candidate model was defined, predicted probabilities of belonging to the compromised category were obtained for each observation in the full DS using the *predict()* function. The classification ability was then evaluated using the area under the curve (**AUC**) of the receiver operating characteristic (**ROC**) curve (Egan, 1975), which plots sensitivity against specificity. Sensitivity referred to the proportion of compromised pigs classified as such by the classification rule, whereas specificity referred to the proportion of normal pigs correctly classified as normal. The ROC curve was drawn, and the AUC was calculated using *roc()* and *auc()* functions of the pROC library (Robin et al., 2011). The candidate model with the highest AUC was selected as the final model.

Based on the ROC curve of the final selected model, a threshold-dependent classification analysis was performed to assess how varying decision thresholds influenced classification metrics. For each threshold (**Ψ**) in (0, 1), predicted probabilities were binarized such that observations with probabilities equal to or exceeding Ψ were classified as compromised, while those below Ψ were classified as normal. A confusion matrix was generated for each Ψ to calculate the numbers of true positives, false positives, true negatives, and false negatives, with compromised pigs considered positives and normal pigs considered negatives (Supplemental Table S1A). Various metrics derived from the confusion matrix were used to evaluate the classification models and identify the optimal Ψ. Among these metrics, accuracy refers to the proportion of pigs correctly classified, whether normal or compromised; the Youden statistic is defined as sensitivity + specificity −1; and positive predictive value (**PPV**) is the proportion of pigs classified as compromised that are truly compromised (Trevethan, 2017), increasing as the number of false positives decreases. In our study, the Ψ based on Youden’s statistic resulted in a high number of false positives, greatly exceeding the number of false negatives. The final Ψ was selected to balance PPV and sensitivity, while also balancing the number of false positives and false negatives, with the justification provided in the Discussion section. The Ψ demonstrating the best overall performance was selected to generate the final predicted classifications up to d 7 of life (Supplemental Table S1B). Predicted values from the final model were visualized using the *plot_model* function from the sjPlot package (Lüdecke, 2024). Predictions were displayed along with their corresponding 95% confidence intervals.

Predictive performance of the final model and threshold was evaluated using *k*-fold cross-validation with *k *= 3, 4, 5, and 10, implemented via the caret package (Kuhn, 2008). The DS was partitioned into *k* folds, with *k -* 1 folds used for training and the remaining fold for validation; this process was repeated *k* times. Performance metrics, accuracy and Cohen’s kappa (which accounts for agreement beyond chance), were calculated for each fold and averaged to provide overall estimates of predictive performance (Supplemental Table S1C).

Additionally, without training the model to weaning, the same model trained to detect compromised pigs on d 7 was used to evaluate its ability to identify compromised pigs at weaning. Therefore, pigs classified as compromised by the non-balanced model at d 7 were followed until weaning. The results of the logistic models gave odds ratios (**OR**) as well as 95% confidence intervals (Fonseca Martinez et al., 2017). The OR represents the change in the odds of the outcome occurring associated with a one-unit increase in the predictor variable, assuming all other variables in the model are held constant.

To evaluate the effect of the “pig category” factor on the most relevant variables identified in the logistic regression model (BW on d 1, RBW on d 1, CI, and parity), beyond differences in mean values, density plots were used to illustrate the full distribution of responses across categories. Sample percentiles (10th, 25th, 50th, 75th, and 90th) were calculated to position cumulative percentages of individuals within the response distribution. Percentiles, which are robust to outliers, indicate the response level below which a given percentage of animals are found (e.g., the 10th percentile, denoted Q10, indicates that 10% of animals fall below this value). To determine if the differences in quantiles were significant, a quantile test (Johnson et al., 1987) was used separately for each pair among the pig categories and each response. This nonparametric approach was implemented in R (Millard, 2013). Statistical significance was accepted at *P *< 0.05 and *P *< 0.10 was considered a trend.

## Results

### Descriptive statistics

The descriptive statistics of farrowing traits and growth performance from birth to weaning in the two DS are presented in Table 3. Regarding farrowing traits, the results differ between DS 1 and DS 2 due to differences in sow prolificacy (hyperprolific sows in DS 1 vs. non-hyperprolific sows in DS 2). The total number of pigs born was higher in DS 1 (mean ± SD: 22.2 ± 4.18) than in DS 2 (mean ± SD: 16.0 ± 4.39). Consequently, DS 2 showed a higher percentage of live born pigs, with an average increase of 2.68% compared to DS 1. Gestation length was the same in both DS, while the number of pigs after cross-fostering differed by an average of 1.5 pigs due to the different cross-fostering protocols followed on each of the farms.

**Table 3.** Descriptive statistics for Dataset 1 and Dataset 2 during the lactation period, including the total number of sows and pigs, mean, and SD

Item	Dataset 1			Dataset 2		
	Total *n*	Mean	SD	Total *n*	Mean	SD
**Farrowing traits (*sows*)**						
** Parity, *n***	102	3.5	2.09	86	4.4	2.41
** Total born, *n***	102	22.2	4.18	86	16.0	4.39
** Live born, %**	102	88.63	8.83	86	91.31	9.37
** Stillborn, %**	102	7.18	7.47	86	7.94	9.04
** Mummified, %**	102	4.19	5.09	86	0.74	2.90
** Gestation length, d**	102	116	0.76	86	116	0.99
** Pigs after CF,^1^ *n***	102	15.0	0.00	86	13.5	1.64
**Growth performance (*pigs*)**						
** BW, kg**						
** Birth**	2,138	1.24	0.32	1,115	1.38	0.36
** D 1**	1,493	1.36	0.33	1,001	1.51	0.37
** D 7**	1,405	2.23	0.60	931	2.64	0.68
** Weaning**	1,336	6.67	1.89	870	5.45	1.44
** ADG, kg**						
** First week, d 0 to 7**	1,405	0.141	0.067	931	0.182	0.079
** D 7 to weaning**	1,336	0.230	0.077	870	0.231	0.077
** Lactation, d 0 to weaning**	1,336	0.206	0.067	870	0.209	0.066

1 Number of pigs per litter after cross-fostering.

ADG = Average daily gain; BW = Body weight.

Regarding BW, pigs in DS 1 had a lower average birth weight (mean ± SD: 1.24 ± 0.32) compared to those in DS 2 (mean ± SD: 1.38 ± 0.36), which is attributed to the higher total number of pigs born per litter in DS 1. This initial difference of 140 g at birth was maintained on d 1 of life and increased to 410 g by d 7, with DS 1 pigs averaging 2.23 ± 0.60 kg and DS 2 pigs 2.64 ± 0.68 kg. However, as pigs in DS 1 experienced an average of 5 additional days of lactation, they ended the lactation period with a 1.22 kg greater BW (mean ± SD: 6.67 ± 1.89 kg) compared to those in DS 2 (mean ± SD: 5.45 ± 1.44 kg). It was observed that the SD increased with age, reflecting greater variability in BW by the end of the lactation period. In terms of ADG, the pattern observed was similar to that of BW. During the first week, ADG was greater in DS 2 (mean ± SD: 0.182 ± 0.079 kg/d) compared to DS 1 (mean ± SD: 0.141 ± 0.067 kg/d). This difference was no longer evident from d 7 to weaning or over the entire lactation period, likely due to the longer lactation duration in DS 1. The mean ADG for the whole lactation period was 0.206 ± 0.067 kg/d in DS 1 and 0.209 ± 0.066 kg/d in DS 2.

### Early-life growth performance variables as predictors of weaning weight

**Figure 1.**
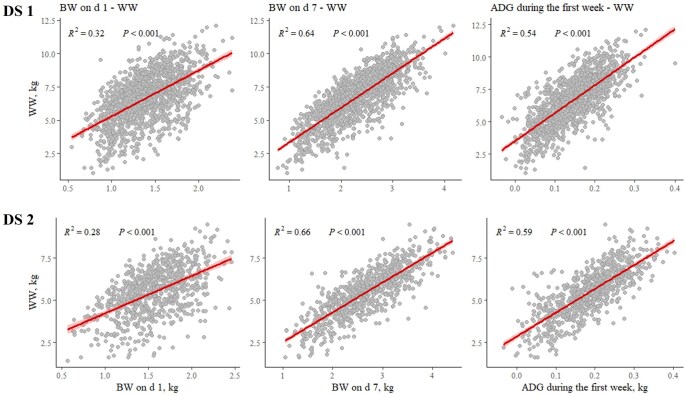
Weaning body weight (WW) in relation to body weight (BW) on d 1, BW on d 7, and average daily gain (ADG) during the first week in Dataset (DS) 1 and DS 2. Each grey dot represents an individual pig. The red line depicts the fitted linear regression model, with the shaded area representing the 95% confidence interval.

All early-life growth performance variables studied showed a positive and significant association with WW (Figure 1). Among them, BW on d 7 emerged as the most powerful predictor of WW, explaining the largest proportion of variability in both DS (*R*^2^ = 0.64 and 0.66 for DS 1 and DS 2, respectively). In contrast, BW on d 1 exhibited the weakest relationship with WW, with lower explanatory power in DS 2 (*R*^2^ = 0.28) than in DS 1 (*R*^2^ = 0.32). Average daily gain during the first week was also strongly associated with WW (*R*^2^ = 0.54 and 0.59 for DS 1 and DS 2, respectively), although its predictive capacity remained below that of BW on d 7. The slightly weaker relationships observed in DS 1 for BW on d 7 and ADG during the first week may be attributed to the longer lactation period (1 wk longer than in DS 2). Nevertheless, both BW on d 7 and ADG during the first week proved to be strong early indicators of weaning performance in both datasets. Although BW on d 1 remained significant, its predictive capacity was notably lower compared with BW on d 7.

### Logistic regression model

**Figure 2.**
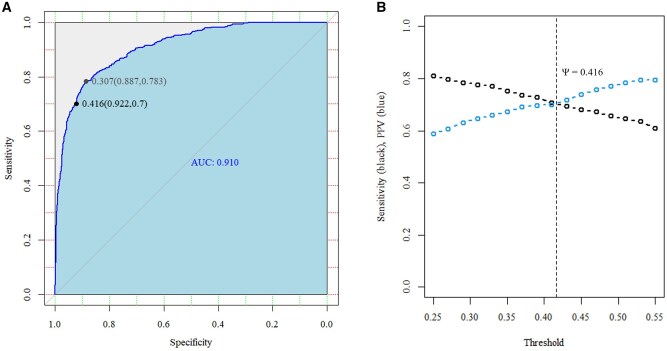
Receiver operating characteristic curve and area under the curve (AUC) for predicting compromised pigs at d 7 of life using a multiple logistic regression model (A). The threshold (Ψ) based on Youden’s statistic was 0.307, whereas balancing sensitivity and positive predictive value (PPV) yielded a Ψ of 0.416. The coordinates of the point associated with each Ψ, including specificity and sensitivity, are shown in parentheses. Sensitivity (black) and PPV (blue) of the multiple logistic regression model across a range of classification thresholds (B). Each point represents the model’s performance at a given Ψ value.

Prediction of compromised pigs on d 7 of life using a binomial logistic regression model achieved an AUC of 0.910 ­(Figure 2A). A final threshold of 0.416 (Figure 2B) was chosen to achieve a balance between sensitivity and PPV, resulting in accuracy = 0.876, sensitivity = 0.700, specificity = 0.922, and PPV = 0.703. On d 7, the model correctly classified 1,726 pigs as normal and 346 as compromised, while misclassifying 146 normal pigs as compromised (false compromised) and 148 compromised pigs as normal (false normal). The confusion matrix and model performance are presented in Supplemental Table S1. Among all pigs classified as compromised by the model, 24.2% died before d 7 and 46.1% had a BW below the 15th percentile of the BW distribution at d 7 (24.2% + 46.1% = 70.3% PPV). Although the model was trained only up to d 7, follow-up of the pigs classified as compromised revealed the following outcomes by weaning: 34.8% died before weaning, and 29.9% had a BW below the 15th percentile of the weaning BW distribution (34.8% + 29.9% = 64.6% PPV). Model performance at weaning was slightly lower than on d 7.

The variables that were significant in the multiple logistic regression model on survivability and growth during the first week of life were BW on d 1 (*P *< 0.001), RBW on d 1 (*P *< 0.001), CI (*P *< 0.001), and parity (*P *= 0.002) (Table 4); all other variables included in the model were not significant. According to the logistic regression model, each 1 kg increase in BW on d 1 was associated with a 96% reduction in the odds of being classified as compromised (OR = 0.04). For a 100 g increase in BW on d 1, the odds of being classified as compromised decreased by approximately 27.6% (results not shown). Similarly, each 1% increase in RBW on d 1 and each 1 g increase in CI were associated with lower odds of being compromised (OR = 0.95 and OR = 0.99, respectively). Parity was positively associated with risk, with each additional parity increasing the odds of a pig being compromised by 10% (OR = 1.10). A significant interaction between BW on d 1 and DS was detected, indicating that the relationship between BW on d 1 and the likelihood of being compromised differed by DS. In DS 1, lower BW on d 1 was associated with a higher probability of being compromised (Estimate = −3.26, *P *< 0.001). In contrast, in DS 2, the negative effect of BW on d 1 was attenuated or even reversed due to the positive interaction term (Estimate = 1.99, *P *= 0.002).

**Table 4.** Multiple logistic regression model evaluating the association between morphological and farrowing traits and the probability of pigs being compromised by day 7 of lactation in terms of survivability and growth performance

Item		Normal	Compromised	Multiple model				
				Estimate	OR	95%-CI		*P*-value
						Low	Up	
**Total**	*n* (%)	1,872 (79.1)	494 (20.9)		–	–	–	–
**Intercept**				3.93				
**BW on d 1, kg**	Mean (SD)	1.52 (0.30)	1.04 (0.26)	−3.26	0.04	0.01	0.15	<0.001
**RBW on d 1, %**	Mean (SD)	6.21 (17.2)	−23.3 (16.9)	−0.05	0.95	0.94	0.97	<0.001
**CI, g/pig**	Mean (SD)	483 (125)	286 (113)	−0.01	0.99	0.99	1.00	<0.001
**Parity, *n***	Mean (SD)	3.84 (2.24)	4.27 (2.33)	0.10	1.10	1.04	1.17	0.002
**Dataset**								
**1 (ref)**	*n* (%)	1,178 (79.9)	297 (20.1)	–	1	–	–	–
**2**	*n* (%)	694 (77.9)	197 (22.1)	−1.78	0.17	0.04	0.80	0.025
**BW on d 1 × Dataset**								
**BW on d 1 × Dataset 2**	–	–	–	1.99	7.30	2.05	26.5	0.002

**Figure 3.**
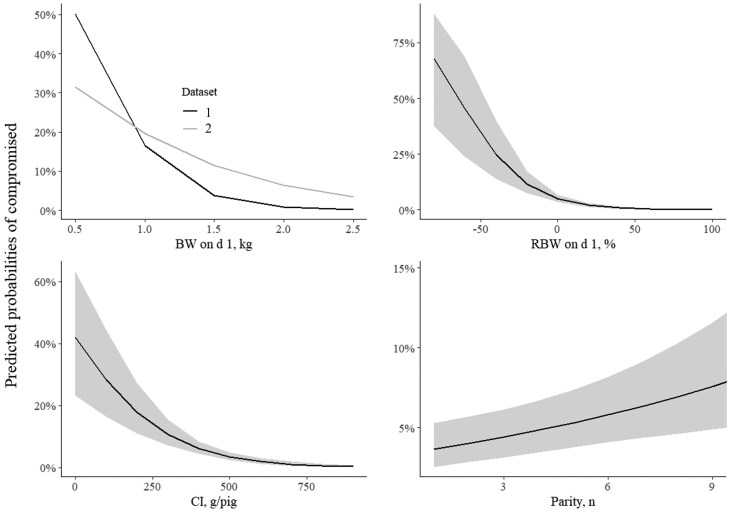
Predicted probabilities of being compromised on d 7 of life as a function of the most relevant variables that significantly influenced this outcome in the logistic regression model. Abbreviations: BW on d 1 = body weight on d 1; RBW on d 1 = relative body weight on d 1.

Figure 3 presents the predicted probabilities of being classified as compromised by d 7 of life based on the significant variables identified in the logistic regression model. For BW on d 1, lower values were associated with a higher risk of being compromised. In DS 1, the probability of compromise was higher below 1 kg compared to DS 2. However, due to the sharp decline in risk observed in DS 1 up to 1 kg, the trend reversed above this threshold, with DS 2 showing a higher probability of compromise beyond 1 kg. This illustrates the significant interaction between BW on d 1 and DS. A strong negative association was also observed for RBW on d 1, where pigs with lower values had a substantially higher risk of being compromised. A value of around 0% RBW on d 1 appeared to be a critical threshold below which the probability of compromise increased to 75%. Regarding CI, the probability of being compromised decreased as intake increased. A threshold of approximately 250 g per pig was associated with a probability of compromise below 20%, with further increases in CI leading to only marginal additional reductions in risk. Although the effect parity was moderate, pigs from older sows tended to have a higher risk of being compromised compared to those from younger sows.

### Probability of early-life risk classification

Based on the odds ratios obtained from the logistic regression model, predicted probabilities of pigs with a BW on d 1 below the 30th percentile being classified as compromised on d 7 were calculated, considering different combinations of BW on d 1, RBW on d 1, and CI, with parity fixed at three (Table 5). Because pigs with low BW on d 1 are at higher risk of being compromised by d 7, this subset of pigs was selected to explore how the other significant variables included in the final model influenced the predicted probabilities. In general, the probability of being compromised decreased as BW on d 1 increased from the 10th to the 30th percentile. Within each BW on d 1 percentile, pigs with low CI and negative RBW on d 1 consistently exhibited the highest risk of being compromised. For example, at the 10th percentile of BW on d 1, a pig with low CI and negative RBW on d 1 had a predicted probability of being compromised exceeding 0.80 in both DS. In contrast, a pig at the same BW on d 1 percentile but with high CI and positive RBW on d 1 showed a lower predicted risk (below 0.25 in both DS). Similar patterns were observed at the 20th and 30th percentiles of BW on d 1. At the 30th percentile, predicted probabilities dropped considerably, particularly for pigs with adequate or high CI and neutral or positive RBW on d 1. The lowest predicted probabilities (0.09 in DS 1 and 0.11 in DS 2) were observed in pigs with high CI and positive RBW on d 1. Notably, the consistency of predictions across both DS supports the robustness of the model.

**Table 5.** Predicted probabilities of pigs below the 30th percentile for body weight on day 1 of life being classified as compromised on day 7, for each dataset

BW on d 1,^1^ kg	CI, ^2^ g/pig	RBW on d 1,^3^ %	Prob. (DS 1)	Prob. (DS 2)
**P10**	Low	Negative	0.87	0.83
		Neutral	0.75	0.73
		Positive	0.63	0.60
	Adequate	Negative	0.72	0.67
		Neutral	0.55	0.53
		Positive	0.41	0.38
	High	Negative	0.51	0.45
		Neutral	0.33	0.31
		Positive	0.22	0.20
**P20**	Low	Negative	0.80	0.77
		Neutral	0.65	0.65
		Positive	0.50	0.50
	Adequate	Negative	0.61	0.58
		Neutral	0.43	0.43
		Positive	0.29	0.29
	High	Negative	0.40	0.36
		Neutral	0.23	0.23
		Positive	0.14	0.14
**P30**	Low	Negative	0.70	0.72
		Neutral	0.52	0.59
		Positive	0.38	0.44
	Adequate	Negative	0.49	0.51
		Neutral	0.31	0.37
		Positive	0.20	0.24
	High	Negative	0.28	0.30
		Neutral	0.15	0.19
		Positive	0.09	0.11

Predictions are based on significant variables identified through multiple logistic regression, with parity fixed at three.

1 BW on d 1 = body weight on day 1 (P10 = 10th percentile; P20 = 20th percentile; P30 = 30th percentile).

2 CI = colostrum intake (adequate = 160 g/kg BW; low = adequate—150; high = adequate + 150).

3 RBW on d 1= relative body weight on day 1 (negative = 25th percentile; neutral = 50th percentile; positive = 75th percentile).

DS = Dataset.

### Quantile analysis of d 1 body weight, relative body weight, colostrum intake, and parity by pig category

**Figure 4.**
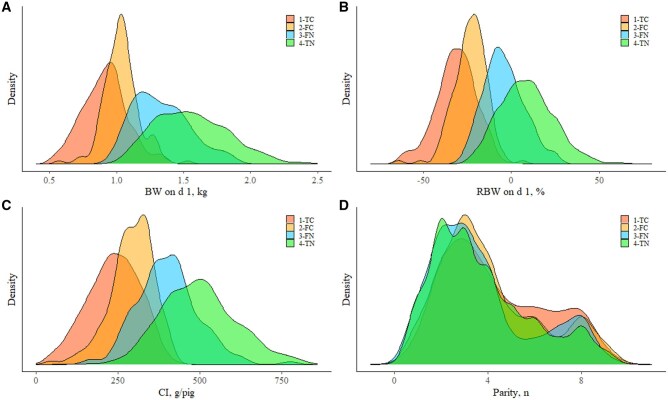
Density plots showing: (A) body weight (BW) on d 1, (B) relative body weight (RBW) on d 1, (C) colostrum intake (CI), and (D) parity for pigs classified into four categories based on the logistic regression model: true compromised (TC, *n* = 346), false compromised (FC, *n* = 146), false normal (FN, *n* = 148), and true normal (TN, *n* = 1,726).

To better understand the pig populations misclassified by the logistic regression model (false compromised and false normal), density plots of BW on d 1, RBW on d 1, CI, and parity across pig categories (true normal, false normal, true compromised, and false compromised) are shown in Figure 4. Statistical details are provided in Supplemental Table S2, and descriptive statistics for all variables included in the model by pig category are presented in Supplemental Table S3. Regarding the well-classified populations, true compromised pigs exhibited a higher density in the left tail, indicating the lowest BW on d 1, RBW on d 1, and CI among all categories. In contrast, true normal pigs showed a higher density in the right tail, reflecting the greatest BW on d 1, RBW on d 1, and CI across all categories. Regarding the misclassified populations, false normal pigs showed significantly higher BW on d 1, RBW on d 1, and CI than false compromised pigs across all evaluated percentiles. This observed shift in density estimates, where false normal pigs outperformed false compromised pigs in key variables included in the model, was the primary reason for misclassification between these two populations.

## Discussion

This study investigated the most influential early-life risk factors affecting growth performance and survival in pigs during the first week of life and throughout lactation. The objective was to develop a multi-criteria model for practical on-farm application, aimed at identifying the optimal timing of interventions to enhance the performance of pigs at higher risk during the lactation period. This is particularly relevant for the pig industry, where uniformity and growth performance are critical, as variability in pig size significantly impacts profitability, with financial, welfare, and environmental consequences (Patience et al., 2004). Body weight variation often comes from a subset of pigs that exhibit markedly slower growth from birth to slaughter, posing significant management challenges and reducing system efficiency (Douglas et al., 2013). Pig performance across the production system results from a complex interaction of factors, with early-life conditions playing a key role in determining lifetime performance (Douglas et al., 2014).

Understanding which early-life conditions most influence subsequent pig performance can help improve survival during the lactation period and increase WW. To identify general indicators of growth performance and survivability, and to ensure the robustness of the proposed model, two independent DS from pig populations with differing levels of prolificacy were characterized and analyzed. The descriptive statistics from both DS were consistent with previous studies (Paredes et al., 2012; Douglas et al., 2013; López-Vergé et al., 2018), supporting the reliability of our data. In line with previous studies suggesting that the predictive value of a factor increases the closer it is to the target weight record (Casellas et al., 2024; Salgado-López et al., 2024), the present results confirm that BW on d 7 and ADG during the first week outperformed BW on d 1 as predictors of WW. These variables therefore serve as strong early indicators of weaning performance in both DS. Additionally, early lactation and the peripartum are when most pre-weaning mortality occurs, with approximately 15% to 20% of all pigs born dying during this time (Baxter and Edwards, 2018; Tucker et al., 2021). This finding is both novel and highly significant, as it demonstrates that events occurring during the first week of life can be as important, or even more important, than birth BW, which has previously been identified as the primary indicator of postnatal performance (Gondret et al., 2005; Rehfeldt et al., 2008; Fix et al., 2010). This may represent a paradigm shift, suggesting that pigs born underweight are not necessarily irreversibly compromised and that early-life interventions in high-risk pigs can improve their subsequent performance. From a practical standpoint, weighing pigs is not a routinely performed on farms. Therefore, producers should recognize the critical importance of the early lactation period rather than focusing solely on birth BW or BW on d 7. Providing targeted care during the first week of life, including ensuring the successful onset of lactation, during which the interaction between the sow and pigs is crucial, has the potential to enhance growth and overall performance up to weaning. Although no interventions during the first week of life were evaluated in this study, the key factors influencing early-life growth performance and survival are discussed below.

While most studies focus on the individual associations between early-life risk factors and pig survival or growth during the preweaning period (Hales et al., 2013; Panzardi et al., 2013; Quesnel et al., 2023) or throughout the production cycle (Paredes et al., 2012; Douglas et al., 2013; He et al., 2016), few publications consider these factors collectively to identify which are most critical to classifying a newborn pig as compromised (low chances of survival and growth) or as normal (Pandolfi et al., 2017; Ward et al., 2020; Geiping et al., 2022). To the authors’ knowledge, no previous studies have proposed a multi-criteria model based on measurable on-farm risk factors within the first 24 h after birth to predict whether a pig is likely to be compromised or normal by d 7 of life. The cut-off weight used to classify a pig as compromised was defined in accordance with previous studies reporting that the incidence of slow-growing pigs on commercial farms ranges from 10% to 15% of the population (Larriestra et al., 2006; He et al., 2016; Calderón Díaz et al., 2017). While other studies have defined slow-growing pigs as those falling within the lower quartile of the BW population distribution (Montoro et al., 2020), a more conservative cut-off was chosen in the present study to avoid excessive inclusion of pigs in the compromised category, thereby reducing the risk of false positives in the model.

The final model on d 7 of life achieved a maximum AUC of 0.910, indicating outstanding discriminatory performance (Hosmer and Lemeshow, 2000). Sensitivity and specificity were adjusted to obtain a balance between predictive values (i.e., PPV) and sensitivity, which is particularly relevant when classifying or screening animals (Fawcett, 2006; Trevethan, 2017). Given that the model based on pre-lactation indicators cannot capture causes of mortality or poor growth that occurs during lactation, it is expected that some cases will not be detected. Therefore, authors accepted a small increase in false negatives in exchange for a non-negligible reduction in false positives. A moderate to high PPV was considered desirable to minimize false positive outcomes from the model, thus supporting the targeted application of specific management strategies (Blavi et al., 2021) or costly supplements (Threadgold et al., 2021) to pigs at highest risk. A PPV of 0.703 indicated that 70.3% of the pigs classified as compromised by the model were indeed truly compromised. When pigs classified as compromised by the model trained only up to d 7 were followed until weaning, the PPV of this model decreased, likely due to the time elapsed between early-life factors and the recording of WW (Casellas et al., 2024; Salgado-López et al., 2024). Nevertheless, a still meaningful PPV of 0.647 was obtained at weaning, indicating that 64.7% of pigs identified as compromised by the early-life model were confirmed as such at weaning.

Poor growth performance and survival during the first week of life and throughout the lactation period were associated with several variables, including BW on d 1, RBW on d 1, CI, and parity. Consistent with the findings of Douglas et al. (2013) and Montoro et al. (2020), who reported that pigs with lower birth weights were able to shift to higher BW categories from birth or weaning to slaughter, our results indicate that BW on d 1 is not the sole determinant of postnatal growth. This also aligns with López-Vergé et al. (2018) and Blavi et al. (2021), who noted that changes in BW category are common during early growth phases, indicating the pig’s potential for compensatory postnatal growth. In terms of survivability, Hales et al. (2013) demonstrated that birth weight alone is not a good indicator of the risk of dying prenatally or shortly after birth. These concepts are supported by the identification of two subsets of pigs (false compromised and false normal) by our model, both of which showed early-life growth performance and survivability independent of their birth characteristics. Specifically, the subset of pigs capable of changing BW category over time had BW on d 1 values ranging from 1.04 ± 0.13 to 1.33 ± 0.21 kg. A similar pattern was observed for RBW on d 1 and CI, with pigs showing RBW on d 1 values between –23.5 ± 9.31% and −4.18 ± 11.3%, and CI ranging from 292 ± 66 to 402 ± 93 g, indicating early-life growth performance that appeared independent of these birth weight-related variables.

Although a proportion of pigs demonstrated the capacity to shift categories during the early growth phases, a negative association between BW on d 1 and the probability of being classified as compromised was observed in the remainder of the population. This finding aligns with numerous studies reporting a positive relationship between higher birth weight and improved survival (Van der Lende and De Jager, 1991; Roehe and Kalm, 2000), with pigs born underweight being at greater risk of mortality (Rehfeldt and Kuhn, 2006) and more likely to remain stunted throughout the production cycle (Quiniou et al., 2002; Gondret et al., 2005; Rehfeldt and Kuhn, 2006). Pigs with a BW below 1 kg on d 1 were at a higher risk of being classified as compromised in litters from hyperprolific sows. This may be attributed to the increased proportion of low-birth-weight pigs (Quiniou et al., 2002; Beaulieu et al., 2010), the decreased amount of colostrum available per pig, and the number of pigs born alive often exceeding the number of functional teats (Bortolozzo et al., 2023). However, appropriate care during the first days of life, particularly through the implementation of targeted cross-fostering protocols, which is a practice especially relevant in large litters (Muns et al., 2016), significantly reduced the risk, especially among pigs with increasing BW on d 1.

Relative weight of pigs is associated with their position within the litter and can therefore be used to identify low-birth-weight individuals. Greater RBW on d 1 values was associated with lower odds of being classified as compromised. In agreement with Milligan et al. (2002a) and Hales et al. (2013), who reported that pigs with low relative birth weight have an increased risk of early mortality, a strong negative association was observed between RBW on d 1 and the predicted probability of being compromised. Relative weight was thus identified as an important indicator of early growth performance and survivability. Similarly, Casellas et al. (2024) found that the difference between BW after cross-fostering and average litter weight was the most important factor in classifying light pigs at weaning using machine learning algorithms. In the present logistic regression model, RBW on d 1 was included instead of BMI, contrasting with the findings of Van der Lende and De Jager (1991) and Hales et al. (2013), who indicated that birth weight and BMI were more reliable predictors of survivability than RBW. Notably, a critical threshold around 0% RBW on d 1 was identified, below which the likelihood of being classified as compromised increased substantially. This highlights the importance of providing immediate care to the lightest pigs in the litter and implementing an appropriate cross-fostering protocol aimed at balancing litter sizes and reducing pre-weaning mortality (Wattanaphansak et al., 2002; Muns et al., 2014).

The RBW of each pig within the litter can be visually assessed on-farm by identifying individuals that is noticeably above or below the average weight of the litter. The identification of these extreme pigs is crucial, as those with the lowest BW are most at risk of pre-weaning mortality (Milligan et al., 2002a, 2002b; Quesnel et al., 2012). Three important recommendations for an effective cross-fostering protocol are: 1) identify viable and non-viable pigs, assigning the smallest ones to the best sows (Muns et al., 2014), 2) ensure that all pigs receive adequate colostrum from their own mother before any movement takes place (Wattanaphansak et al., 2002), and 3) minimize the number and frequency of movements, trying to keep the maximum number of pigs with their own mother (Wattanaphansak et al., 2002). Based on these recommendations and our results, the authors highlight the importance of fostering the smallest pigs to achieve low variation within their litters, without the need to minimize within-litter variation across all litters. This approach would allow farmers to keep pigs with their birth mothers as much as possible.

Colostrum consumption significantly contributes to the survival and development of pigs, providing immunoglobulins for immune protection as well as energy for thermoregulation and growth (Devillers et al., 2004). In this study, we observed a reduction in the odds of being classified as compromised with each unit increase in CI, highlighting the critical importance of adequate colostrum consumption for pig survival after birth and growth until weaning, as also noted by Quesnel et al. (2023). Interestingly, a threshold of approximately 250 g per pig was associated with a probability of being classified as compromised below 20%, with further increases in intake yielding only marginal additional reductions in risk. This finding aligns with the recommendation by Quesnel et al. (2012), who suggested that pigs should consume at least 250 g of colostrum within the first 24 h to ensure survival. Other authors have proposed an intake of 150 to 170 g/kg BW as the minimum required to ensure neonatal survival, particularly in low-birth-weight pigs (Le Dividich et al., 2005; De Vos et al., 2014). In hyperprolific sows, prolonged farrowing duration combined with intense litter competition at the udder may reduce the amount of colostrum ingested and delay its intake after the onset of parturition (Oliviero et al., 2019). In such farrowings, reduced vitality immediately after birth can also significantly impair pigs’ ability to ingest colostrum (Oliviero et al., 2019), which is one of the most important factors influencing their initial suckling behavior (Peltoniemi et al., 2021). A 27 g CI difference in favor of DS 2 compared to DS 1 aligns with these results regarding hyperprolific sows. Thus, ensuring sufficient CI for survival until weaning remains a major challenge, especially for low-birth-weight pigs born to hyperprolific sows.

Colostrum intake can be easily assessed on-farm by palpating the abdomen to determine fullness (Geiping et al., 2022). Several management strategies can be implemented to support proper colostrum consumption. Oral supplementation with colostrum recently milked from sows within the same herd has been shown to increase immunoglobulin G blood levels in weaker pigs, promoting better thermoregulation and improving overall litter performance within the first 24-h (Muns et al., 2014). Additionally, split suckling (Rutherford et al., 2013; Solà-Oriol and Gasa, 2017), performed just prior to cross-fostering, has proven effective in ensuring CI in less vital pigs, resulting in improved daily weight gain and greater litter homogeneity at weaning (Donovan and Dritz, 1996).

Parity was the final significant variable included in the logistic regression model. In this study, each additional parity increased the odds of a pig being classified as compromised by 10%, which is in accordance with Roehe and Kalm (2000), who reported a greater risk of mortality among pigs born to sows of parity 2 to 5 compared to those born to parity 1 sows. Similarly, other studies have shown increased pig mortality and a higher prevalence of crushing with increasing parity (Roehe and Kalm, 2000; Weber et al., 2009). Douglas et al. (2013) also identified parity as a significant factor for growth in all periods of pig life, although the pattern across the consecutive parities was unclear. In the same way, several authors have highlighted parity number as a key determinant of pig performance, with pigs born to gilts being lighter at birth than those born to sows (Smith et al., 2007; Quesnel et al., 2008). In contrast, Wulbers-Mindermann et al. (2002) and Hales et al. (2013) reported a greater risk of mortality among pigs born to primiparous sows compared to those born from multiparous sows. Additionally, the effect of parity on colostrum yield, and consequently on early-life growth performance, appears inconsistent across studies (Quesnel and Farmer, 2019; Quesnel et al., 2023).

The results were consistent across the two DS, providing confidence that the conclusions drawn may have broader applicability. This consistency highlights the potential utility of the model as a predictive tool for the early identification of pigs at risk. Nonetheless, it is important to note that the inclusion of DS as a significant fixed effect in the final model may limit the generalizability of the results to other DS. If a new DS shares the same descriptive characteristics as those used to develop the model, DS can be treated as a fixed effect. Conversely, if the new DS differs in its descriptive characteristics, it may be more appropriate to include DS as a random effect, which could influence some of the predictive performance of the final model. Another limitation of the model is that it was not validated using an independent third DS, which could have further strengthened the generalizability of the findings. Nevertheless, predictive performance was rigorously assessed through *k*-fold cross-validation, yielding high accuracy and substantial agreement, thereby supporting the model’s robustness and reliability.

When focusing on pigs inherently at higher risk (BW on d 1 below the 30th percentile), the model demonstrated how early postnatal factors can either exacerbate or mitigate this vulnerability. As expected, greater BW on d 1 was associated with a general decline in the predicted probability of being classified as compromised, confirming the strong association between greater BW on d 1 and improved survival and growth performance identified in the logistic regression model. However, even within each BW on d 1 percentile group, the influence of RBW on d 1 and CI remained evident. Pigs with poor CI and a negative position within the litter consistently showed the highest predicted risk, underscoring the critical importance of both CI and relative BW position within the litter, particularly among the most vulnerable individuals. In contrast, pigs with high CI and a positive RBW on d 1 exhibited significantly lower predicted risks. These findings suggest that early-life interventions aimed at enhancing CI and promoting immediate postnatal growth may be effective in reducing early-life risk, even in pigs with low-birthweights.

Farmers could benefit from using the variables identified in the logistic regression model as a guideline for determining when to implement interventions and which pigs to target, thereby enhancing their future growth performance and survival. This approach is particularly relevant in hyperprolific populations, where increased prolificacy often leads to a significant reduction in average birth weight, resulting in a higher number of low-birth-weight pigs (Quiniou et al., 2002; Beaulieu et al., 2010). In the swine industry, the greatest proportion of BW variability originates at birth, increasing slightly during lactation and in the first days post weaning until pigs adapt to solid feed (López-Vergé et al., 2018). From this point until slaughter, variability tends to decrease, mainly due to routine management practices implemented on farms (López-Vergé et al., 2018). The lactation period represents the most appropriate window of opportunity to implement strategies that enable lighter pigs to successfully catch up to their heavier pen mates, thereby reducing BW variability within the population.

## Conclusions

This study provides a better understanding of key early-life indicators associated with pig survival and postnatal growth from birth to weaning under commercial conditions. Among the early growth performance variables analyzed, body weight at d 7 and average daily gain during the first week emerged as the strongest predictors of weaning weight, underscoring the critical importance of early lactation for subsequent postnatal performance. Using logistic regression, a model with good predictive ability was developed to identify pigs at risk of poor growth or mortality by d 7 of life, based on body weight on d 1, relative body weight on d 1, colostrum intake, and sow parity. The consistency of model’s performance across two independent populations, despite differences in farm management and prolificacy, supports the generalizability and robustness of this risk classification approach. Integrating these risk factors into a predictive framework may offer swine producers a valuable tool to target interventions more precisely during the neonatal period.

## Supplementary Material

skaf402_Supplementary_Data

## Abbreviations

ADG: average daily gain
AIC: Akaike Information Criterion
AUC: area under the curve
BMI: body mass index
BW: body weight
CI: colostrum intake
CRL: crown-to-rump length
DS: dataset
OR: odds ratio
PI: ponderal index
PPV: positive predictive value
RBW: relative body weight
ROC: receiver operating characteristic
SID: standardized ileal digestible
VIF: variance inflation factor
WW: weaning weight
