# Supplementary material for: Early-life risk factors predicting growth retardation and mortality in pigs: a multi-criteria approach
Source: J Anim Sci. 2025 Nov 19;104:skaf402. doi: 10.1093/jas/skaf402 (PMC12959324; doi:10.1093/jas/skaf402)
Supplement: skaf402_Supplementary_Data [file skaf402_supplementary_data.docx]

| **Supplemental Table 1. (A)** Confusion matrix comparing the predicted results and real conditions of the proposed model trained up to day 7. **(B)** Model performance metrics (Accuracy, Sensitivity, Specificity, and Positive Predictive Value) across different thresholds. **(C)** Accuracy and Cohen’s kappa obtained from *k*-fold cross-validation. | | | | | | | | | |
| --- | --- | --- | --- | --- | --- | --- | --- | --- | --- |
| **A)** | | | | | | | | | |
| **Day 7 Model** | Total population = 2,366 | | | True condition | | | | | |
|  |  |  |  | 1 | | | 0 | | |
| Predicted condition | 1 | | | 346 | | | 146 | | |
|  | 0 | | | 148 | | | 1,726 | | |
| **B)** |  | | |  | | |  | | |
| **Threshold criterion** | Threshold | Accuracy^1^ | | Se^2^ | | Sp^3^ | | | PPV^4^ |
| Youden statistic^5^ | 0.307 | 0.866 | | 0.783 | | 0.887 | | | 0.647 |
| Se ≈ PPV | 0.416 | 0.876 | | 0.700 | | 0.922 | | | 0.703 |
| **C)** |  | | |  | | |  | | |
| **Cross-validation folds** | 3-fold | | 4-fold | | 5-fold | | | 10-fold | |
| Accuracy | 0.891 | | 0.886 | | 0.889 | | | 0.890 | |
| Cohen’s kappa^6^ | 0.648 | | 0.634 | | 0.641 | | | 0.646 | |
| ^1^Accuracy = (TC + TN) / n = (346 + 1,726) / 2,366.  ^2^Se = Sensitivity = TC / (TC + FN) = 346 / (346 + 148).  ^3^Sp = Specificity = TN / (TN + FC) = 1,726 / (1,726 + 146).  ^4^PPV = Positive Predictive Value = TC / (TC + FC) = 346 / (346 + 146).  ^5^Youden statistic = Se + Sp – 1.  ^6^Cohen’s kappa = measure of agreement beyond chance, calculated as (P_o_ – P_e_) / (1 – P_e_), where P_o_ is the observed agreement and P_e_ the agreement expected by chance.  Abbreviations: FN = False normal; FC = False compromised; TN = True normal; TC = True compromised. | | | | | | | | | |

| **Supplemental Table 2.** Effect of the predicted category from the logistic regression model on quantile change (Q10, Q25, Q50, Q75, and Q90) according to body weight on day 1 **(A)**, relative body weight on day 1 **(B)**, colostrum intake **(C)**, and parity **(D)**. The reference groups used in this analysis are the True normal (TN), True compromised (TC), and False compromised (FC). | | | | | | | | | | |
| --- | --- | --- | --- | --- | --- | --- | --- | --- | --- | --- |
| **A)** | | | | | | | | | | |
| **Item** | **Q10** | ***P* Q10** | **Q25** | ***P* Q25** | **Q50** | ***P* Q50** | **Q75** | ***P* Q75** | **Q90** | ***P* Q90** |
| TN | 1.22 | < 0.001 | 1.34 | < 0.001 | 1.54 | < 0.001 | 1.75 | < 0.001 | 1.92 | < 0.001 |
| FN | 1.1 |  | 1.17 |  | 1.31 |  | 1.46 |  | 1.6 |  |
| TN | 1.22 | < 0.001 | 1.34 | < 0.001 | 1.54 | < 0.001 | 1.75 | < 0.001 | 1.92 | < 0.001 |
| FC | 0.89 |  | 0.956 |  | 1.03 |  | 1.1 |  | 1.19 |  |
| TC | 0.695 | < 0.001 | 0.8 | < 0.001 | 0.915 | < 0.001 | 1 | < 0.001 | 1.12 | 0.004 |
| FC | 0.89 |  | 0.956 |  | 1.03 |  | 1.1 |  | 1.19 |  |
| TC | 0.695 | < 0.001 | 0.8 | < 0.001 | 0.915 | < 0.001 | 1 | < 0.001 | 1.12 | < 0.001 |
| FN | 1.1 |  | 1.17 |  | 1.31 |  | 1.46 |  | 1.6 |  |
| FC | 0.89 | < 0.001 | 0.956 | < 0.001 | 1.03 | < 0.001 | 1.1 | < 0.001 | 1.19 | < 0.001 |
| FN | 1.1 |  | 1.17 |  | 1.31 |  | 1.46 |  | 1.6 |  |
| **B)** |  |  |  |  |  |  |  |  |  |  |
| **Item** | **Q10** | ***P* Q10** | **Q25** | ***P* Q25** | **Q50** | ***P* Q50** | **Q75** | ***P* Q75** | **Q90** | ***P* Q90** |
| TN | -10.3 | < 0.001 | -2.11 | < 0.001 | 8.06 | < 0.001 | 18.3 | < 0.001 | 28.1 | < 0.001 |
| FN | -17.9 |  | -11.8 |  | -5.57 |  | 2.45 |  | 11.8 |  |
| TN | -10.3 | < 0.001 | -2.11 | < 0.001 | 8.06 | < 0.001 | 18.3 | < 0.001 | 28.1 | < 0.001 |
| FC | -35 |  | -28.6 |  | -22.8 |  | -17.7 |  | -13.3 |  |
| TC | -46.1 | < 0.001 | -38.2 | < 0.001 | -30.9 | < 0.001 | -24 | < 0.001 | -17 | 0.001 |
| FC | -35 |  | -28.6 |  | -22.8 |  | -17.7 |  | -13.3 |  |
| TC | -46.1 | < 0.001 | -38.2 | < 0.001 | -30.9 | < 0.001 | -24 | < 0.001 | -17 | < 0.001 |
| FN | -17.9 |  | -11.8 |  | -5.57 |  | 2.45 |  | 11.8 |  |
| FC | -35 | < 0.001 | -38.6 | < 0.001 | -22.8 | < 0.001 | -17.7 | < 0.001 | -13.3 | < 0.001 |
| FN | -17.9 |  | -11.8 |  | -5.57 |  | 2.45 |  | 11.8 |  |
| **C)** | | | | | | | | | | |
| **Item** | **Q10** | ***P* Q10** | **Q25** | ***P* Q25** | **Q50** | ***P* Q50** | **Q75** | ***P* Q75** | **Q90** | ***P* Q90** |
| TN | 355 | < 0.001 | 417 | < 0.001 | 495 | < 0.001 | 570 | < 0.001 | 655 | < 0.001 |
| FN | 288 |  | 347 |  | 402 |  | 448 |  | 524 |  |
| TN | 355 | < 0.001 | 417 | < 0.001 | 495 | < 0.001 | 570 | < 0.001 | 655 | < 0.001 |
| FC | 205 |  | 258 |  | 298 |  | 338 |  | 367 |  |
| TC | 126 | < 0.001 | 183 | < 0.001 | 238 | < 0.001 | 294 | < 0.001 | 338 | 0.001 |
| FC | 205 |  | 258 |  | 298 |  | 338 |  | 367 |  |
| TC | 126 | < 0.001 | 183 | < 0.001 | 238 | < 0.001 | 294 | < 0.001 | 338 | < 0.001 |
| FN | 288 |  | 347 |  | 402 |  | 448 |  | 524 |  |
| FC | 205 | < 0.001 | 258 | < 0.001 | 298 | < 0.001 | 338 | < 0.001 | 367 | < 0.001 |
| FN | 288 |  | 347 |  | 402 |  | 448 |  | 524 |  |
| **D)** | | | | | | | | | | |
| **Item** | **Q10** | ***P* Q10** | **Q25** | ***P* Q25** | **Q50** | ***P* Q50** | **Q75** | ***P* Q75** | **Q90** | ***P* Q90** |
| TN | 1 | 0.503 | 2 | 0.526 | 3 | 0.361 | 5 | 0.672 | 7.5 | 0.834 |
| FN | 1 |  | 2 |  | 3 |  | 5 |  | 8 |  |
| TN | 1 | 0.947 | 2 | 0.995 | 3 | 0.889 | 5 | 0.818 | 7.5 | 0.848 |
| FC | 2 |  | 3 |  | 3.5 |  | 5 |  | 8 |  |
| TC | 2 | 0.953 | 3 | 0.705 | 4 | 0.888 | 6 | 0.963 | 8 | 0.776 |
| FC | 2 |  | 3 |  | 3.5 |  | 5 |  | 8 |  |
| TC | 2 | 0.999 | 3 | 0.997 | 4 | 0.995 | 6 | 0.870 | 8 | 0.792 |
| FN | 1 |  | 2 |  | 3 |  | 5 |  | 8 |  |
| FC | 2 | 0.920 | 3 | 0.977 | 3.5 | 0.899 | 5 | 0.684 | 8 | 0.588 |
| FN | 1 |  | 2 |  | 3 |  | 5 |  | 8 |  |
| Abbreviations: FN = False normal; *P* = *P*-value. | | | | | | | | | | |

| **Supplemental Table 3.** Descriptive statistics (mean, SD, minimum, maximum) for pigs classified into four categories based on the logistic regression model: True compromised (n = 346), False compromised (n = 146), False normal (n = 148), and True normal (n = 1,726). | | | | | | | | | | | | | | | | | | | |
| --- | --- | --- | --- | --- | --- | --- | --- | --- | --- | --- | --- | --- | --- | --- | --- | --- | --- | --- | --- |
| **Item** | **True compromised** | | | |  | **False compromised** | | | |  | **False normal** | | | |  | **True normal** | | | |
|  | **Mean** | **SD** | **Min.** | **Max.** |  | **Mean** | **SD** | **Min.** | **Max.** |  | **Mean** | **SD** | **Min.** | **Max.** |  | **Mean** | **SD** | **Min.** | **Max.** |
| Significant variables |  |  |  |  |  |  |  |  |  |  |  |  |  |  |  |  |  |  |  |
| BW on d 1, kg | 0.91 | 0.17 | 0.50 | 1.38 |  | 1.04 | 0.13 | 0.57 | 1.53 |  | 1.33 | 0.21 | 0.99 | 1.84 |  | 1.56 | 0.27 | 0.91 | 2.46 |
| RBW on d 1, % | -31.6 | 11.4 | -64.3 | 1.55 |  | -23.5 | 9.31 | -64.2 | 6.36 |  | -4.18 | 11.3 | -27.8 | 25.0 |  | 8.72 | 15.2 | -28.1 | 82.5 |
| CI, g/pig | 237 | 79 | 29 | 433 |  | 292 | 66 | 46 | 416 |  | 402 | 93 | 165 | 776 |  | 499 | 115 | 144 | 845 |
| Parity, n | 4.5 | 2.3 | 1 | 9 |  | 4.1 | 2.1 | 1 | 9 |  | 3.8 | 2.4 | 1 | 9 |  | 3.8 | 2.2 | 1 | 9 |
| Pig additional variables |  |  |  |  |  |  |  |  |  |  |  |  |  |  |  |  |  |  |  |
| Traits at birth |  |  |  |  |  |  |  |  |  |  |  |  |  |  |  |  |  |  |  |
| CRL, cm | 21.0 | 1.48 | 18.1 | 26.0 |  | 21.5 | 1.28 | 18.4 | 26.0 |  | 23.3 | 1.94 | 14.4 | 28.0 |  | 24.1 | 1.69 | 18.5 | 32.0 |
| BMI, kg/m^2^ | 20.6 | 2.50 | 13.9 | 27.6 |  | 21.6 | 2.25 | 14.2 | 29.3 |  | 23.1 | 2.88 | 16.6 | 37.4 |  | 24.7 | 2.85 | 15.0 | 38.9 |
| PI, kg/m^3^ | 98.6 | 13.7 | 60.8 | 130.5 |  | 100.6 | 12.6 | 57.8 | 138.5 |  | 100.4 | 19.3 | 65.5 | 241.1 |  | 102.8 | 14.0 | 51.7 | 210.1 |
| RBW on d 0, % | -26.3 | 12.2 | -60.2 | 17.4 |  | -20.5 | 10.8 | -62.9 | 25.8 |  | -1.85 | 13.5 | -40.5 | 43.2 |  | 9.39 | 15.8 | -33.6 | 87.2 |
| RT, ºC | 35.8 | 1.6 | 32.1 | 40.4 |  | 36.2 | 1.7 | 32.6 | 39.8 |  | 36.4 | 1.3 | 33.0 | 39.0 |  | 36.8 | 1.4 | 32.0 | 40.6 |
| Vitality ≥ 2, % | 38.0 | - | - | - |  | 44.8 | - | - | - |  | 44.4 | - | - | - |  | 42.1 | - | - | - |
| BW on d 0, kg | 0.91 | 0.17 | 0.54 | 1.35 |  | 1.01 | 0.15 | 0.55 | 1.58 |  | 1.26 | 0.22 | 0.72 | 1.81 |  | 1.44 | 0.26 | 0.79 | 2.44 |
| Performance after birth |  |  |  |  |  |  |  |  |  |  |  |  |  |  |  |  |  |  |  |
| BW gain 24h, g | 1.85 | 61.6 | -200 | 160 |  | 28.5 | 59.4 | -300 | 130 |  | 69.7 | 78.3 | -180 | 450 |  | 114 | 75.7 | -310 | 360 |
| Abbreviations: BMI = Body mass index; BW = Body weight; CI = Colostrum intake; CRL = Crown-to-rump length; PI = Ponderal index; RBW = Relative body weight; RT = Rectal temperature. | | | | | | | | | | | | | | | | | | | |
